# Systematic genetic analysis of pediatric patients with autoinflammatory diseases

**DOI:** 10.3389/fgene.2023.1065907

**Published:** 2023-01-27

**Authors:** Yvonne Poker, Sandra von Hardenberg, Winfried Hofmann, Ming Tang, Ulrich Baumann, Nicolaus Schwerk, Martin Wetzke, Viola Lindenthal, Bernd Auber, Brigitte Schlegelberger, Hagen Ott, Philipp von Bismarck, Dorothee Viemann, Frank Dressler, Christian Klemann, Anke Katharina Bergmann

**Affiliations:** ^1^ Department of Human Genetics, Hannover Medical School, Hannover, Germany; ^2^ L3S Research Center, Leibniz University Hannover, Hannover, Germany; ^3^ Department of Pediatric Pneumology, Allergology and Neonatology, Hannover Medical School, Hannover, Germany; ^4^ Department of Pediatrics and Pediatric Hematology/Oncology, University Children’s Hospital, Oldenburg, Germany; ^5^ Division of Pediatric Dermatology, Children’s Hospital Auf der Bult, Hannover, Germany; ^6^ Department of Pediatrics, University Medical Center Schleswig‐Holstein, Campus Kiel, Kiel, Germany; ^7^ Translational Pediatrics, Department of Pediatrics, University Hospital Würzburg, Würzburg, Germany

**Keywords:** autoinflammatory diseases, inborn errors of immunity (IEI), FMF, whole exome sequencing (WES), genetic diagnostics

## Abstract

Monogenic autoinflammatory diseases (AID) encompass a growing group of inborn errors of the innate immune system causing unprovoked or exaggerated systemic inflammation. Diagnosis of monogenic AID requires an accurate description of the patients’ phenotype, and the identification of highly penetrant genetic variants in single genes is pivotal. We performed whole exome sequencing (WES) of 125 pediatric patients with suspected monogenic AID in a routine genetic diagnostic setting. Datasets were analyzed in a step-wise approach to identify the most feasible diagnostic strategy. First, we analyzed a virtual gene panel including 13 genes associated with known AID and, if no genetic diagnosis was established, we then analyzed a virtual panel including 542 genes published by the International Union of Immunological Societies associated including all known inborn error of immunity (IEI). Subsequently, WES data was analyzed without pre-filtering for known AID/IEI genes. Analyzing 13 genes yielded a definite diagnosis in 16.0% (*n* = 20). The diagnostic yield was increased by analyzing 542 genes to 20.8% (*n* = 26). Importantly, expanding the analysis to WES data did not increase the diagnostic yield in our cohort, neither in single WES analysis, nor in trio-WES analysis. The study highlights that the cost- and time-saving analysis of virtual gene panels is sufficient to rapidly confirm the differential diagnosis in pediatric patients with AID. WES data or trio-WES data analysis as a first-tier diagnostic analysis in patients with suspected monogenic AID is of limited benefit.

## 1 Introduction

Autoinflammatory diseases (AID) are caused by dysregulation of the innate immune system with heterogeneous symptoms that occur during recurrent episodes of unprovoked inflammation (McDermott et al., 1999; Ben-Chetrit et al., 2018). This inflammation can be caused by genetic alterations in immunoregulative genes. AIDs are therefore, considered as Inborn Errors of Immunity (IEI), which are categorized by the International Union of Immunological Societies (IUIS) into 10 subgroups with distinctive features (Tangye et al., 2021). However, due to the heterogeneous clinical presentation of AID, diseases belonging to other IEI subtypes should be taken into consideration as differential diagnosis for AID. Next-generation sequencing is a powerful diagnostic tool in this process. We aim to support the most feasible strategy for the genetic diagnostic work-up of patients with suspicion of AID. The most common monogenic AID are Familial Mediterranean fever (FMF), cryopyrin-associated periodic syndromes, TNF receptor-associated periodic syndrome, deficiency of IL-1 receptor antagonist, and hyper-IgD syndrome (Ciccarelli et al., 2014). In contrast, PFAPA (periodic fever, aphthous stomatitis, pharyngitis and cervical adenitis) and unclassified systemic autoinflammatory diseases (SAID) are groups without known monogenic etiopathology (Thomas et al., 1999; Lachmann, 2011; Ter Haar et al., 2019). Clinically, autoinflammation can present with fever, arthritis, mucositis, serositis, musculoskeletal and nervous system involvement as well as skin symptoms. Clinical consensus criteria for initiating genetic testing are not clearly defined. A genetically confirmed diagnosis enables tailored treatment, prognostic estimation of disease process and familial risk estimates. On the other hand, exclusion of an underlying monogenic cause in certain cases also allows to optimize clinical classification and empirical treatment.

Still over half of all patients presenting with AID do not undergo genetic diagnostics (Elferink et al., 2015; Papa et al., 2020). Initiating genetic testing in AID usually depends on the treating physician’s evaluation, experience and general understanding of genetics. In addition, the decision raises ethical and psychosocial issues, and it can be complex to interpret the results. Informing patients of their genetic status and helping patients and families deal with the possible consequences is time-consuming (Pasquier et al., 2021). Therefore, it is essential to identify patients that should be prioritized.

Analyzing whole exome/genome sequencing (WES/WGS) data, instead of gene panels has become a diagnostic routine in diagnostic laboratories due to its technological advancements and growing experience. Moreover, WES/WGS data can be reevaluated, and availability of new clinical information could lead to more conclusive genetic diagnosis, as shown in other disease entities (Basel-Salmon et al., 2019). To decipher clinical relevance, phenotypic correlation and optimal comprehension of genetic testing in AID, we conducted a step-by-step analysis of 125 unrelated children with a clinical presentation of AID. We analyzed different virtual gene panels based on WES as well as complete WES or WGS data and correlated these with the clinical and phenotypic presentation.

## 2 Methods and materials

### 2.1 Patient cohort

The study was conducted in accordance with the principles outlined in the Declaration of Helsinki (World Medical Association, 2013) and approved by the Ethics Committee of Hanover Medical School (ID 9849_BO_K_2021). The analyzed cohort included 125 pediatric index patients diagnosed with suspected monogenic AID who were transferred for genetic testing between 2018 and 2021. The following inclusion criteria had to be met: age of the patient <18 years, evidence of systemic inflammation (fever/autoinflammatory symptoms that persists for prolonged periods of several months to years with or without elevated inflammatory parameters (Material and Methods 2.3) before initiation of therapy), the suspicion of a likely monogenic disease, e.g., early onset of the disease or familial inheritance and/or exclusion of recurrent infection (Touitou et al., 2019).

### 2.2 Sample preparation, whole-exome sequencing and data analysis

WES was performed in all 125 enrolled patients, as well as in 36 parents as part of trio-WES. Sample preparation, sequencing, and data analysis are described in the Supplementary Material.

### 2.3 WES analysis

First, WES data was screened with a virtual gene panel. For the virtual gene panel 1 (vPANEL_1), we chose 13 genes with reported pathogenic variants causative for an AID, namely *IL1RN*, *IL36RN*, *LPIN2*, *MEFV*, *MVK*, *NLRC4*, *NLRP12*, *NLRP3*, *NOD2*, *PLCG2*, *PSMB8, PSTPIP1* and *TNFRSF1A*. For patients that remained without a definitive diagnosis, we used a second virtual gene panel (vPANEL_2). This includes 542 genes published by the IUIS Committee as causative for IEI (Bousfiha et al., 2020; Pasquier et al., 2021) and genes which are associated with the HPO terms “recurrent fever” or/and “increased inflammation” (Robinson et al., 2008). All analyzed genes are listed in Supplementary Table S1. Further information (e.g., filter strategies) can be found in Supplementary Material.

### 2.4 Blood parameters for inflammatory markers

The treating physicians provided clinical and laboratory data (if available). Blood parameters for inflammatory markers were compiled for each patient, which were preferably measured before initiation of therapy. In total, no laboratory data were reported at all for 6 patients (ID40, ID41, ID49, ID56, ID63, ID84) (Supplementary Table S2). Levels of CRP (C-reactive protein, reference <5 mg/L) and ESR (erythrocyte sedimentation rate, reference <20 mm/h) were documented, as well as serum calprotectin (reference <2.9 μg/ml), serum amyloid A (reference <6.4 mg/L), and blood count (Supplementary Table S2).

## 3 Results

### 3.1 Detailed characteristics of patients with AID

The mean age of the patients at genetic testing was 6.8 years (4 days–18 years). The total cohort consisted of 60 (48%) female and 65 (52%) male patients. In 12 families (9.6%), consanguinity (first- or second-degree cousins) was self-reported. Detailed patient characteristics including ethnicity and age of onset are listed in Supplementary Table S3. On average, the latency between disease manifestation and genetic workup was 3 years (0.1–14.5 years). Hospitalization was required in 61 patients (48.8%) due to their underlying inflammatory disease. Seven patients (5.6%) needed ICU treatment, including two with a fatal outcome (patients ID23 and ID48). To define the patient phenotypes according to the Human Phenotype Ontology (HPO) (Robinson et al., 2008), we classified the symptoms of AID presentation according to the 15 main functional systems as follows: body temperature regulation, gastrointestinal tract (GI tract), musculoskeletal system, skin barrier, mucous membranes, visual system, effusion, lymphatic system, susceptibility to infection, respiratory system, cardiovascular system, kidney and urinary tract, auditory system, nervous system, and laboratory biomarkers positive for systemic inflammation. The top six functional system phenotypes were: 1. body temperature regulation (*n* = 92, 73.6%), 2. positive laboratory biomarkers (*n* = 86, 68.8%), 3. GI tract (*n* = 54, 43.2%), 4. musculoskeletal system (*n* = 40, 32%), 5. lymphatic system and skin barrier, each reported in 35 patients (28%).

### 3.2 Deciphering genetic alterations in AID

**FIGURE 1:**
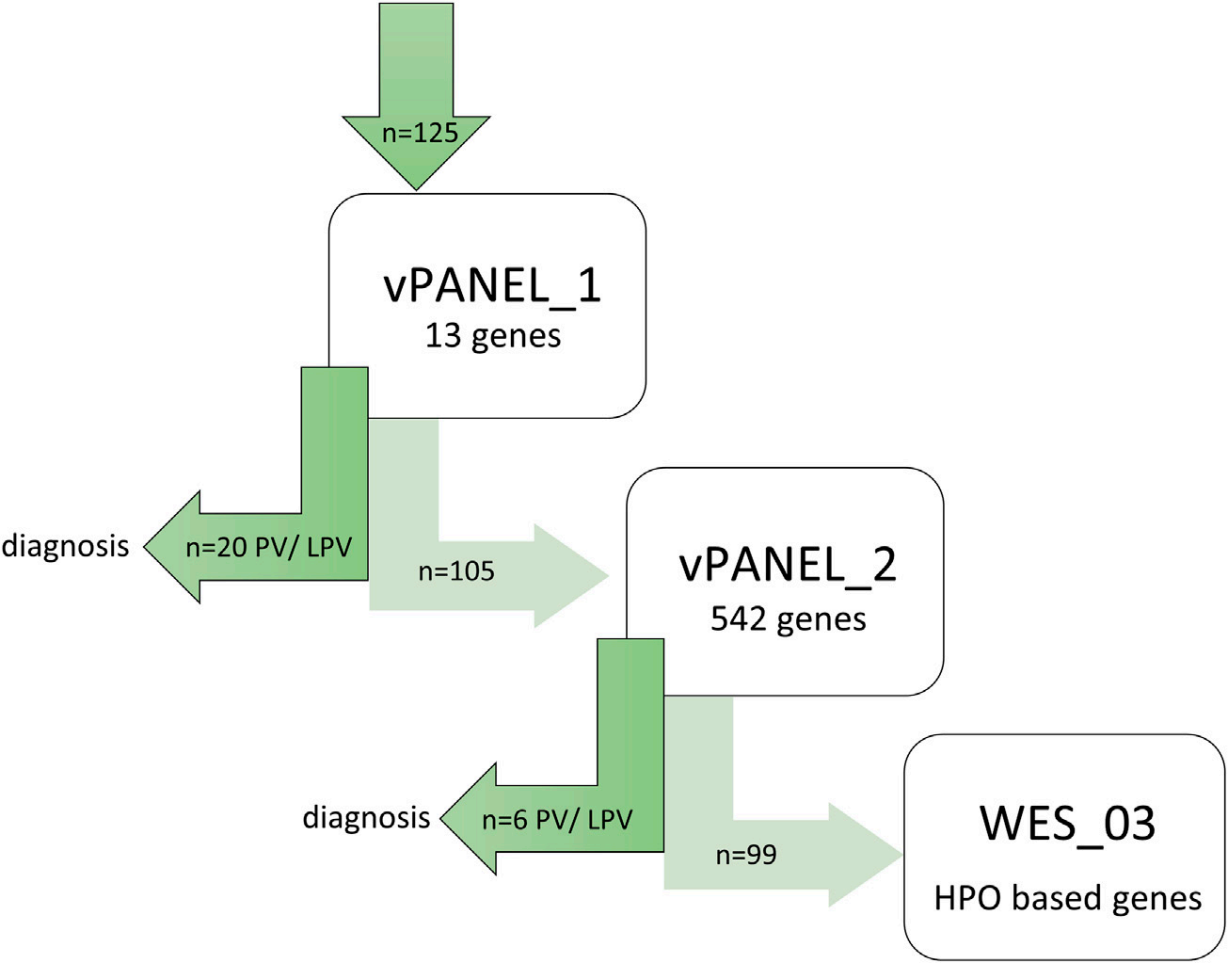
Genetic workflow displaying step-by-step diagnostic strategy. First, WES data from all patients were screened using virtual vPANEL_1 (13 genes); in case of non-confirmatory findings, virtual vPANEL_2 was analyzed (542 genes). In a third step, WES data (WES_3) were filtered to detect the most likely deleterious variants highly suspicious for AID. Additionally, HPO terms were used according to the patient’s phenotype. If applicable, trio WES were performed; PV = pathogenic, or LPV = likely pathogenic variant found through genetic diagnostics.

Patients were screened step-by-step for vPANEL_1, vPANEL_2 or WES_3 (see above and Figure 1) to determine to what extent genetic testing was needed. Applying testing with vPANEL_1, pathogenic variants (PV) or likely pathogenic variants (LPV) were identified in 20/125 individuals (16.0%) in the genes *MEFV* (*n* = 19) and *NLRP3* (*n* = 1) (Table 1). 25 variants of unknown significance (VUS, class 3) were identified in 30/125 patients (24%) (Supplementary Table S4). Applying testing with vPANEL_2, PVs or LPVs were identified in 6/125 individuals (4.8%) in *ADA2*, *CYBB*, *IL10RB*, *MS4A1*, *RIPK1* and *TTC37* (each *n* = 1) (Supplementary Material). 11 VUS were identified in 11/125 patients (11.2%) (Supplementary Table S4). Overall, monoallelic LPV were identified in four genes known to follow an autosomal recessive inheritance (*CIITA*, *NCKAP1L*, *MVK*, *POLE2*), but a second variant was not detected (Supplementary Table S4). Likewise, analyzing CNVs in these regions yielded no segmental gains or losses in DNA sequence. VUS should not be used in clinical decision-making yet, but monitoring for the disorder in question was recommended.

**TABLE 1:** Pathogenic variants identified by vPANEL_1.

ID patient	Gene	Coding DNA (c.) protein (p.)	Coding DNA (c.) protein (p.)	Zygosity	Mode of inheritance	Class	Transcript	Phenotype (MIM) presenting in patient
		Allele 1	Allele 2					
2	*MEFV*	c.2080A>G p.(Met694Val)	c.2080A>G p.(Met694Val)	hom	AR, AD	5	NM_000243.2	FMF AD (MIM134610) FMF, AR (MIM249100)	
3	*MEFV*	c.2080A>G p.(Met694Val)	c.2080A>G p.(Met694Val)	hom	AR, AD	5			
5	*MEFV*	c.2080A>G p.(Met694Val)	c.2080A>G p.(Met694Val)	hom	AR, AD	5			
6	*MEFV*	c.2080A>G p.(Met694Val)	c.2080A>G p.(Met694Val)	hom	AR, AD	5			
13	*MEFV*	c.2080A>G p.(Met694Val)	c.2080A>G p.(Met694Val)	hom	AR, AD	5			
14	*MEFV*	c.2080A>G p.(Met694Val)	c.2080A>G p.(Met694Val)	hom	AR, AD	5			
17	*MEFV*	c.2040G>C p.(Met680Ile)	c.2040G>C p.(Met680Ile)	hom	AR, AD	5			
4	*MEFV*	c.2040G>C p.(Met680Ile)	c.2282G>A p.(Arg761His)	comp-het	AR, AD	5/4			
10	*MEFV*	c.2040G>C p.(Met680Ile)	c.2282G>A p.(Arg761His)	comp-het	AR, AD	5/4			
1	*MEFV*	c.2080A>G p.(Met694Val)	—	het	AR, AD	5			
11	*MEFV*	c.2080A>G p.(Met694Val)	—	het	AR, AD	5			
15	*MEFV*	c.2080A>G p.(Met694Val)	—	het	AR, AD	5			
31	*MEFV*	c.2082A>G p.(Met694Ile)	—	het	AR, AD	5			
12	*MEFV*	c.2177T>C p.(Val726Ala)	—	het	AR, AD	5			
7	*MEFV*	c.2040G>C p.(Met680Ile)	-	het	AR, AD	5			
8	*MEFV*	c.2040G>C p.(Met680Ile)	—	het	AR, AD	5			
16	*MEFV*	c.2040G>C p.(Met680Ile)	—	het	AR, AD	5			
125	*MEFV*	c.2040G>C p.(Met680Ile)	—	het	AR, AD	5			
62	*MEFV*	c.2177T>C p.(Val726Ala)	—	het	AR, AD	5			
22	*NLRP3*	c.1043C>T p.(Thr348Met)	—	het	AD	5	NM__001079821.2	CINCA syndrome (MIM607115) Muckle-Wells syndrome (MIM191900)	
19	*ADA2*	c.139G>A p.(Gly47Arg)	c.139G>A p.(Gly47Arg)	hom	AR	5	NM_001282225.2	Vasculitis, autoinflammation, immunodeficiency, and hematologic defects syndrome (MIM615688)	
23	*CYBB*	c.1511G>C p.(Gly504Ala)	—	hemi	XLR	4	NM_000397.3	Chronic granulomatous disease (MIM306400)	
18	*IL10RB*	c.477G>A p.(Trp159*)	c.477G>A p.(Trp159*)	hom	AR	4	NM_000628.4	Inflammatory bowel disease 25 (MIM612567)	
55	*MS4A1*	c.203dup p.(Leu69Serfs*72)	—	het	AR	4	NM_152866.2	Immunodeficiency, common variable, 5 (MIM613495)	
25	*RIPK1*	c.970G>C p.(Asp324His)	—	het	AD	5	NM_001354930.1	Autoinflammation with episodic fever and lymphadenopathy (MIM618852)	
20	*TTC37*	c. 2453_2454delTG p.(Val818Glufs*5)	c.(-228 + 1216_-228 + 1222)_ (326 + 92_ 326 + 98) p.?	comp-het	AR	4/4	NM_014639.3	Tricho-hepato-enteric syndrome 1 (MIM222470)	

For the remaining 99 patients, WES data (WES_3) was filtered to detect the most likely deleterious variants highly suspicious for AID. Additionally, HPO terms were used according to the patient’s phenotype. A brief literature review was performed on each gene to evaluate clinical significance and its role in IEI, especially in AID and the inflammasome. 15 VUS, highly suspicious for AID, were identified in 14 genes in 13 patients (*ALPK1*, *AXL*, *BAHD1*, *CARD10*, *IGF2R*, *IRGM*, *LTK*, *MAPK14*, *NFKBIL1*, *PLA2R1*, *RHBDF1*, *RNF220*, *TRIM21*, and *ZC3H12A*) (Supplementary Table S5). Some of the identified genes (e.g., *ALPK1*, *IRGM* and *NFKBIL1*) are linked to a specific phenotype in OMIM (OMIM, 2021). VUS not fulfilling the aforementioned criteria are not listed and need further evaluation. Trio-WES was initially requested in 17 patients (13.2%) to achieve a genetic result in critically ill children. Segregation analysis to evaluate suspicious variants found in singleton WES was performed using Sanger sequencing (*n* = 14) and WES (*n* = 1). In order to exclude an underlying second genetic disease in the patients who had already received a genetic diagnosis through vPANEL_1 or vPANEL_2, these patients were finally also examined using WES_3. None of these further evaluations identified a clear PV.

### 3.3 Expanding differential diagnosis through genetic testing in suspected AID

**FIGURE 2:**
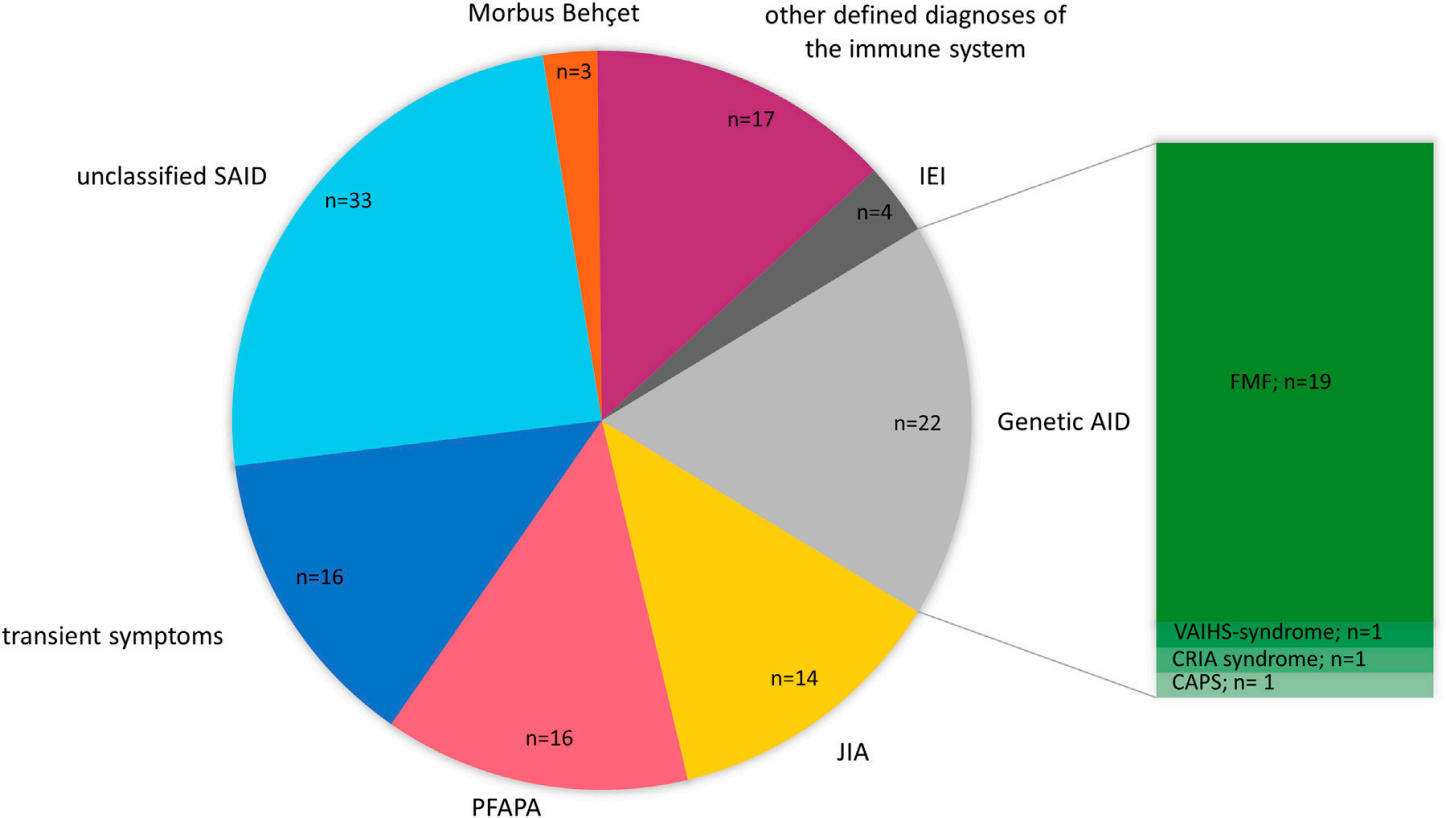
Final diagnosis of pediatric AID cohort after genetic testing. The pie chart is presenting the final diagnosis of 125 pediatric patients with AID categorized into the following subclasses: Genetic autoinflammation (AID), juvenile idiopathic arthritis (JIA), PFAPA, patients with transient symptoms, unclassified SAID, Mobus Behçet, other defined disease of the immune system (no genetic cause identified) and Inborn Errors of Immunity (IEI) (other than autoinflammation). AID was subdivided into FMF, VAIHS-syndrome, CAPS and CRIA syndrome. n = amount of patients.

We initially sequenced all patients in our cohort with a suspicion of an AID. In some patients, however, the genetic results and following clinical reassessment confirmed a final clinical diagnosis that belongs to a subgroup of IEI other than AID. Therefore, we categorized the patients’ final clinical diagnosis into the following subclasses: genetic autoinflammation (AID) (*n* = 22), patients with transient symptoms (*n* = 16), juvenile idiopathic arthritis (JIA) including systemic JIA (SJIA) (*n* = 14), PFAPA (*n* = 16), unclassified SAID (*n* = 33), Morbus Behçet (*n* = 3), defined disease of the immune system without known genetic cause (*n* = 17), and IEI_other than autoinflammation (IEI) (*n* = 4). AID was subdivided into FMF (*n* = 19), VAIHS-syndrome (*ADA2*; *n* = 1), CAPS (*NLRP3*; *n* = 1) and CRIA-syndrome (*RIPK1*; *n* = 1). IEI other than AID was subdivided into Inflammatory bowel disease 25 (*IL10RB*; *n* = 1), chronic granulomatous disease (CYBB; *n* = 1), tricho-hepato-enteric syndrome 1 (*TTC37*; *n* = 1) and CVID with hypogammaglobulinemia (*MS4A1*; *n* = 1) (Figure 2).

### 3.4 Correlation of laboratory biomarkers and PVs in the MEFV gene

**FIGURE 3:**
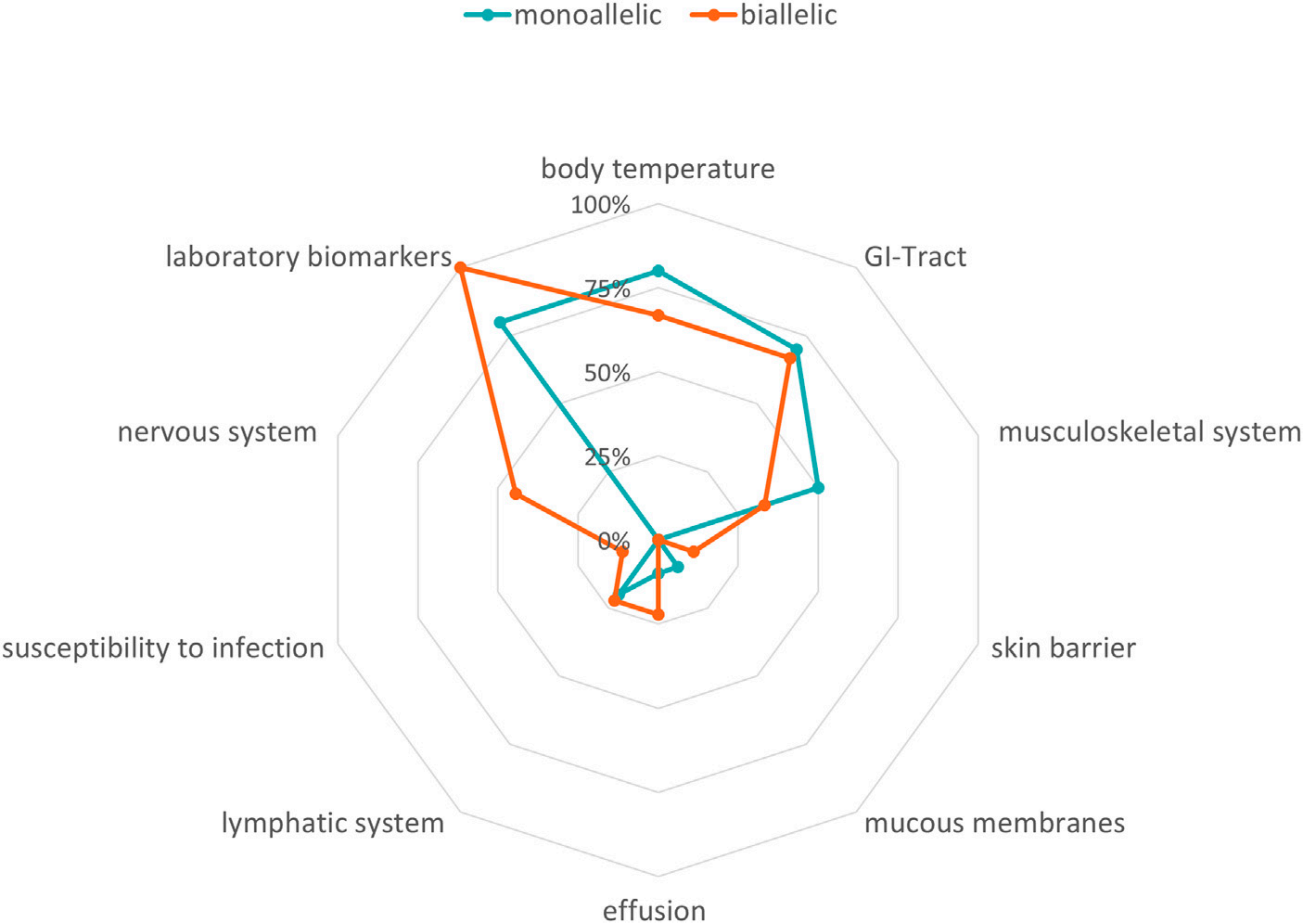
Phenotypes in FMF. Radar chart visualizing the symptoms of biallelic and monoallelic carriers of pathogenic *MEFV* variants depending on zygosity. Turquoise line: amount of patients with monoallelic variants in %, orange line: amount of patients with biallelic variants in %.

**FIGURE 4:**
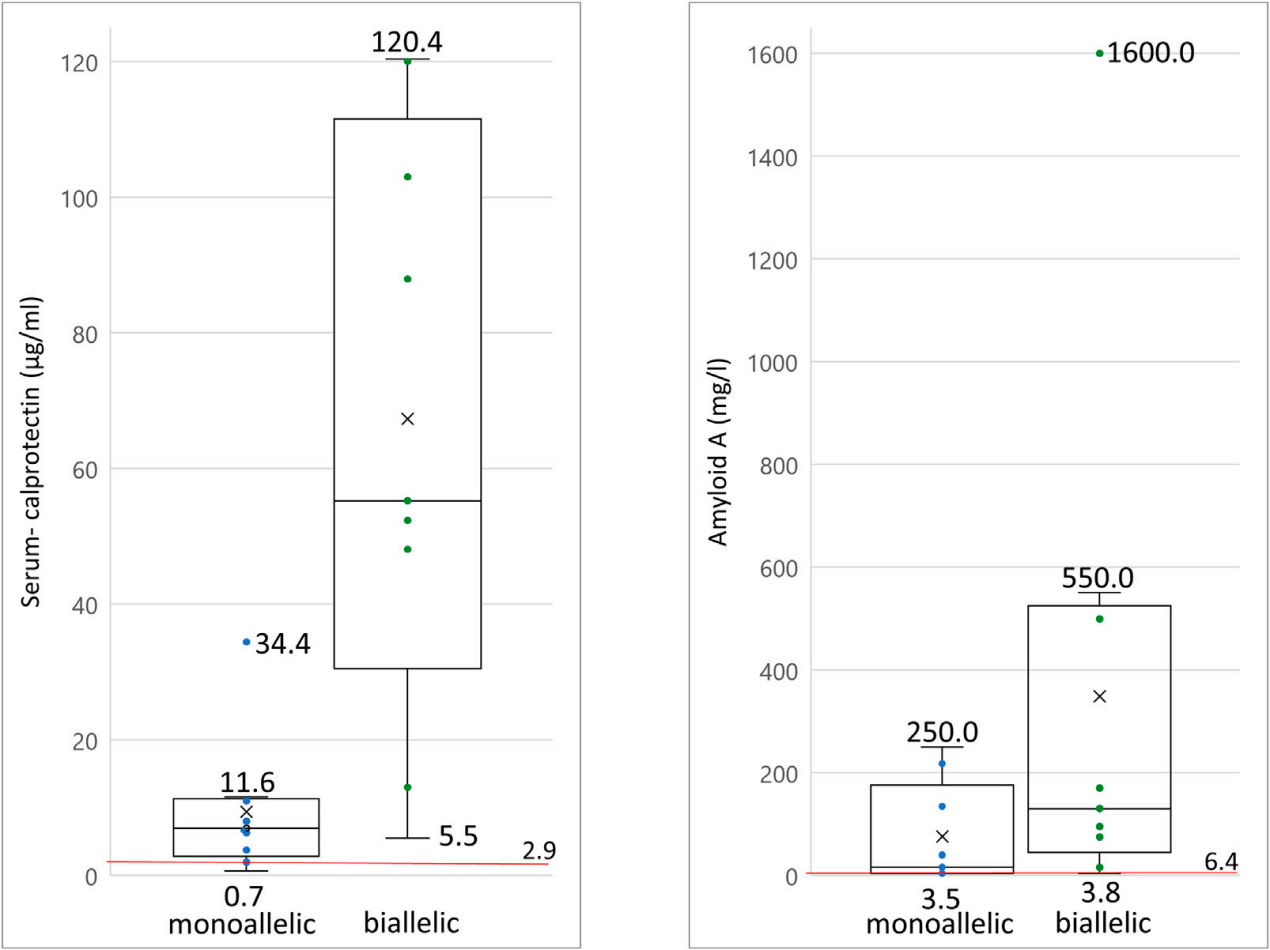
Serum levels of inflammatory biomarkers measured in patients with pathogenic MEFV variants a) serum calprotectin levels of monoallelic and biallelic carriers of pathogenic *MEFV* variants; b) serum amyloid A levels of monoallelic and biallelic carriers of pathogenic *MEFV* variant. Each dot and each horizontal extreme line corresponds to a value in one patient (n = 9); cross symbolizes the mean value of all patients; red line drawn at reference serum level cut-off point; the horizontal line in the middle of a box plot displays the median.

Biallelic PVs in the *MEFV* gene confirm the clinical diagnosis of FMF, although heterozygous individuals have also been shown to exhibit classical disease-related symptoms (French FMF Consortium, 1997; Padeh and Berkun, 2016). In our cohort, 19/125 patients carried at least one PV in the *MEFV* gene (Table 1). Laboratory biomarkers showed signs of inflammation in 100% (*n* = 9) of the patients carrying biallelic PVs in *MEFV* and 80% (*n* = 8) of the patients carrying monoallelic PVs (Figure 3, Supplementary Table S2). Differences in other affected functional systems between biallelic and monoallelic carriers of *MEFV* PVs are shown in Figure 3. Blood levels of serum calprotectin, CRP, ESR, and serum amyloid A may be of value to monitor disease course and the patient’s response to treatment (Berkun et al., 2007). The differences became obvious when we compared these laboratory biomarkers of patients with biallelic PVs in *MEFV* with those with monoallelic PVs in *MEFV*. Median serum calprotectin was 55.2 μg/ml in the group of biallelic carriers (*n* = 9) and 7.0 μg/ml in the group of monoallelic carriers (*n* = 9) (Student’s t-test: *p* < 0.001, ref value < 2.9 μg/ml). Considering serum amyloid A, the median level in the group of biallelic carriers was 130 mg/L, whereas the median level in the group of monoallelic carriers was 16.0 mg/L (Student’s t-test: *p* = 0.07, ref value 6.4 mg/L) (Figure 4, Supplementary Table S2).

## 4 Discussion

A monogenic AID diagnosis is based on a careful interpretation of the clinical phenotype and results of molecular genetic analysis. We performed genetic testing in a cohort of 125 pediatric patients with suspected AID and addressed the use of different virtual panels and WES data. In the first approach, vPANEL_1 yielded a clear pathogenic variant (PV) in 16.0% of patients. Analyzing the remaining patients by vPANEL_2 led to an additional positive genetic result in 4.8%. Remarkably, in 3 of these 26 patients, an IEI different to AID was diagnosed based on genetic results (*IL10RB* defect, chronic granulomatous disease, and tricho-hepato-enteric syndrome). In our cohort, WES data analysis did not establish a reliable diagnosis of the inflammatory phenotype. Furthermore, trio-WES did not increase our diagnostic yield.

In summary, the overall diagnostic yield in our cohort was 20.8%. In addition, we identified VUS in 43.2%, which could become clinically relevant in the future. This rate supports other studies focusing on the genetic diagnosis of AID (Thomas et al., 1999; Yao et al., 2016; Hoang and Albert, 2019; Karacan et al., 2019; Batlle-Masó et al., 2020; Demir et al., 2020; Okano et al., 2020). Cohorts of equal size (Thomas et al., 1999; Ter Haar et al., 2019; Okano et al., 2020) and larger cohorts (Yao et al., 2016) have both been evaluated in these studies, and some have only included pediatric patients (World Medical Association, 2013; Fathalla et al., 2021; Kosukcu et al., 2021), while others have also included adults (Yao et al., 2016; Omoyinmi et al., 2017; Hoang and Albert, 2019; Karacan et al., 2019; Batlle-Masó et al., 2020; Okano et al., 2020). A higher positive yield has been reported in studies with a more selective choice in the patient cohort (e.g. diagnostic yield of 36% in cohorts with frequent consanguinity (Kosukcu et al., 2021)). Studies focusing on unclassified SAIDs usually have shown low diagnostic rates (Demir et al., 2020). The term “unclassified SAIDs” has been used to describe patients who do not meet the clinical diagnostic criteria for an AID or have clinical findings for more than one AID (Thomas et al., 1999; Harrison et al., 2016). In our study, we ranked 30 patients, for whom a definitive diagnosis of a monogenic disease could not be made, in the “unclassified SAIDs” category. Among these, 8 patients had VUS in *LPIN2*, *TNFAIP3*, *MEFV*, *NLRC4*, *NLRP1* or *NLRP3*, which may contribute to their phenotype.

Confirmatory testing for phasing variants in recessive disorders and the determination of inheritance using Sanger sequencing led to an upgrading (ID20, ID22, ID23, ID25) or downgrading (ID39, ID51, ID55, ID59, ID68, ID77, ID107, ID109) of their pathogenicity classification. Systematic segregation analyses have not been included in recent reports. Moreover, in contrast to other studies (Thomas et al., 1999; Yao et al., 2016; Omoyinmi et al., 2017; Hoang and Albert, 2019; Karacan et al., 2019; Batlle-Masó et al., 2020; Kosukcu et al., 2021), we included trio-WES, which is traditionally considered superior for genetic disease diagnosis and the determination of *de novo* occurrence often upgrading the pathogenicity classification of variants. Furthermore trio-WES normally decreases turnaround time for positive and negative cases (Kingsmore et al., 2019), therefore, yielding a quicker diagnosis with a synopsis of parental and offspring genetic data. The average of the diagnostic yields reported in several studies focusing on trio-WES on heterogeneous pediatric populations with any kind of suspected genetic disease was collected and reviewed by Dragojlovic et al. They state that there is a diagnostic yield of 34.4% (SD = 5.9%, *n* = 13) for trio-WES and 26.6% (SD = 8.0%, *n* = 7) for single WES (Dragojlovic et al., 2018). In contrast, in our cohort which includes patients with a precisely defined phenotype of AID, trio-WES did not yield additional clearly pathogenic results. A likely explanation is the difference in our stringent patient selection criteria in contrast to children with any suspected genetic disorder. In the 16 patients with suspected PFAPA, virtual panel diagnostics could not confirm a monogenic cause alteration. However, PFAPA syndrome is a clinical diagnosis with different classification criteria (Marshall et al., 1987; Tasher et al., 2006; Gattorno et al., 2008; Cochard et al., 2010; Ciccarelli et al., 2014; Dragojlovic et al., 2018). Since a relevant number of patients with monogenic periodic fever syndromes also meet the diagnostic criteria for PFAPA syndrome (Thomas et al., 1999; Tasher et al., 2006), failure to diagnose patients with very similar phenotypes could be fatal. Gattorno et al. have demonstrated that 83% of patients with mevalonate kinase deficiency, 57% of patients with TRAPS, and 8% of patients with FMF have met the criteria for PFAPA syndrome (Gattorno et al., 2009). Hofer et al. have postulated that the exclusion of monogenic periodic fever syndromes, e.g., by ruling out pathogenic variants in the *MEFV* gene, should be included in a new version of the criteria for PFAPA syndrome (Hofer et al., 2014). A high allele frequency of *MEFV* variants (27%–66%) has been reported previously in PFAPA patients (Celiksoy et al., 2016). Therefore, PFAPA syndrome is not unexpected in children with *MEFV* variants. The underlying pathogenesis of PFAPA syndrome and the genotype-phenotype correlation in *MEFV* variants are both not fully understood. This suggests the presence of additional underlying factors determining the phenotype in heterozygous carriers of *MEFV* variants. Patient ID62, for example, who presented with symptoms pointing towards PFAPA syndrome was diagnosed with heterozygous *MEFV* PV and successfully treated with prednisolone. Based on the Thomas criteria (Thomas et al., 1999) and following monogenic disease exclusion, 16 patients (ID63 to ID78) in our cohort could be diagnosed with PFAPA syndrome.

There are some limitations to this study. Laboratory testing for inflammatory markers was not measured for all and not equally in all cases at the first consultation. We recommend a standardized laboratory determination for further studies. Another limitation is that Trio WES was not available for every patient. This study includes a large group of pediatric patients with different ethnicities.

**FIGURE 5:**
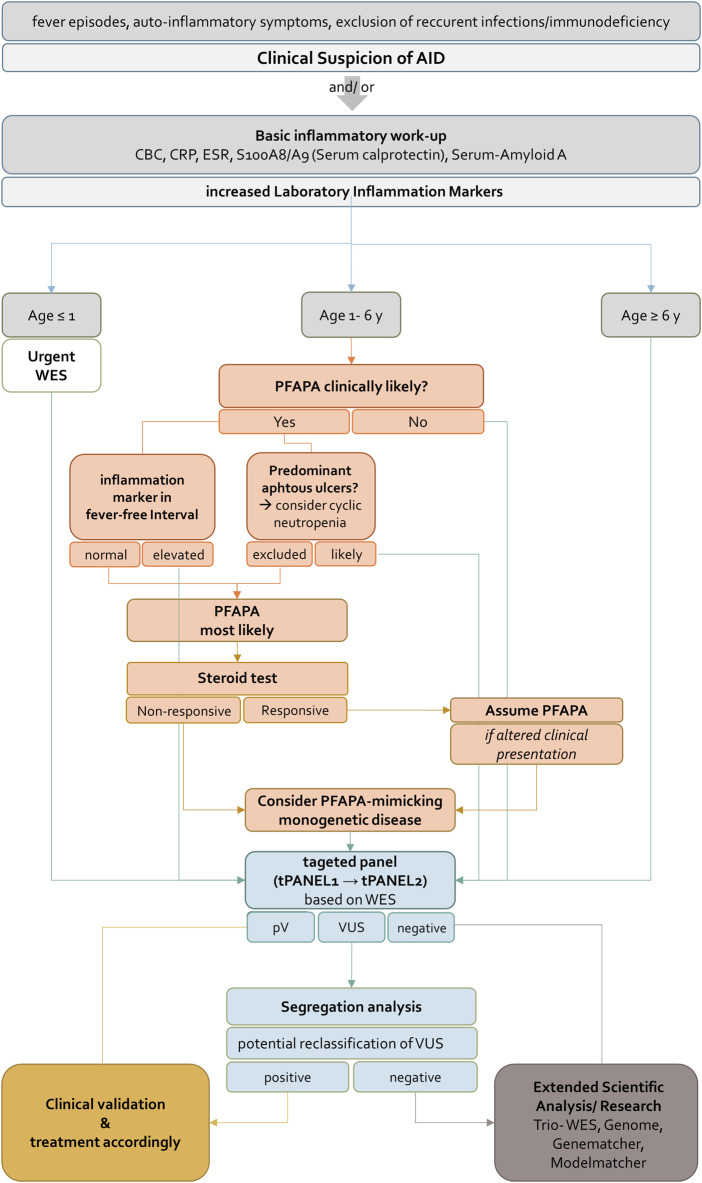
Flowchart for diagnosis, treatment and genetic evaluation of patients with suspected AID. AID should be suspected if patients present with fever episodes and auto-inflammatory signs and symptoms. If CBC, CRP, ESR, S100A8/A9 (Serum calprotectin) and Serum-Amyloid A are increased the further approach depends on the age of the patient. For patients younger than 1 year or older than 6 years WES should be requested immediately. According to clinical guidelines patients between one and 6 years PFAPA syndrome should be ruled out in a first step. If inflammation markers in fever-free intervals are not elevated and cyclic neutropenia is excluded in patients with oral ulcers, PFAPA syndrome is most likely. A response to a steroid test confirms the diagnosis. A non-responsive steroid test indicates a PFAPA-mimicking monogenetic disease and WES should be performed. For all children identification of pathogenic variant(s) in tPANEL entails clinical validation and treatment according to the disease. For negative sequencing results an extended scientific analysis (e.g. Trio- WES, Genome, analysis of genes of uncertain significance) is recommended. Variants of unknown significance can be further evaluated by segregation analysis. Confirmation of variants as having arisen *de novo* may result in reclassification under ACMG guidelines. AID, autoinflammatory disease, CBC, complete blood count, CRP, C-reactive protein, ESR, erythrocyte sedimentation rate, WES, whole exome sequencing, PFAPA, Periodic Fever, Aphthous Stomatitis, Pharyngitis, Adenitis, pV, pathogenic variant, VUS, Variant of unknown significance.

To date, clinical consensus criteria for initiating genetic testing in patients with suspected AID are not clearly defined. To reduce diagnostic costs and time, we present this stepwise genetic testing approach to be used in clinically stable patients based on WES data. Even in patients meeting clinical diagnostic criteria for specific AID analysis of underlying genetics might be beneficial regarding evaluation of responds to certain treatments, disease severity and complications as mentioned in a recent study on 500 cases with FMF (Beshlawy et al., 2022). Many patients in our cohort were diagnosed by analyzing a small set of 13 genes specific to common AID (tPANEL1). Expanding this set of genes to all genes previously described in human errors of immunity (tPANEL2) has been effective in identifying additional PVs. Explorative analysis of whole exome data without filtering to known genes did not offer further diagnostic yield in this study. Nevertheless, in a research setting WES and trio-WES analysis will result in the identification of yet undescribed genes or VUS, which could become clinically relevant in the future. This is especially true for complex phenotypes where autoinflammation only accounts for part of the symptoms. In summary, we integrated the appropriate genetic diagnostic test into the clinical decision-making process to develop an integrative approach for genetic diagnostics in children presenting with clinical suspicion of AID (Figure 5).

## Data Availability

The original data presented in the study are included in the article. Further inquiries can be made directly to the corresponding author. The datasets for this article are not publicly available due to concerns regarding participant/patient anonymity. Requests to access the datasets should be directed to the corresponding author.

## Abbreviations

AID, Autoinflammatory diseases; CRP, C-reactive protein; ESR, erythrocyte sedimentation rate; FMF, familial mediterranean fever; GI tract, gastrointestinal tract; HPO, human phenotype ontology; IEI, inborn error of immunity; IUIS, international union of immunological societies; JIA, juvenile idiopathic arthritis; LPV, likely pathogenic variants; OMIM, online mendelian inheritance in man; PV, pathogenic variants; PFAPA, periodic fever, aphthous stomatitis, pharyngitis and cervical adenitis; SAID, systemic autoinflammatory diseases; SJIA, systemic JIA; TRAPS, TNF receptor-associated periodic syndrome; VUS, variants of unknown significance; WES, whole exome sequencing; WGS, whole genome sequencing.
