# Supplementary material for: Systematic genetic analysis of pediatric patients with autoinflammatory diseases
Source: Front Genet. 2023 Jan 27;14:1065907. doi: 10.3389/fgene.2023.1065907 (PMC9911692; doi:10.3389/fgene.2023.1065907)
Supplement: Supplementary file 2 [file Table1.DOCX]

Supplementary Material

**Material and Methods**

Sample preparation and whole exome sequencing

DNA was extracted from blood or buccal mucosa smears using NucleoMag Blood kit (Macherey-Nagel, Düren, Germany). DNA quality was verified by measuring the DNA with the Qubit® Fluorometer using the Qubit dsDNA BR Assay Kit (Life Technologies, Darmstadt, Germany). WES was performed using the IDT Exome library kit (xGen, IDT, Leuven, Belgium). Captured DNA was sequenced using Illumina NextSeq 500 Sequencer to generate 150‐bp paired‐end reads. To verify the DNA sample, 14 single nucleotide polymorphisms (SNP) were amplified by means of competitive allele-specific PCR using fluorescence-labeled primers and analyzed by StepOnePlus software for genotyping experiments (StepOnePlus System, Thermo Fisher Scientific, Inc., Waltham, MA, USA). SNP results were compared with the data from the NGS analysis (megSAP). Reads were aligned to the human reference genome (UCSC Genome Browser build GRCh37/hg19). A mean coverage of 150 reads with 98.5% of bases within the targeted region covered by at least 20 reads was achieved. Variants that were localized in the intron region (except +/- 20) as well as variants with low mapping quality were excluded. To keep only rare variants, we barred variants with an allele frequency >0.1% in the overall population of Exome Aggregation Consortium19 (ExAC), the 1000 Genomes Project, and the Genome Aggregation Database (gnomAD). In order to get information about the variants’ pathogenicity, we used prediction tools, including phyloP, SIFT, PolyPhen-2, FATHMM, and CADD. In addition, LOVD, ClinVar, and gnomAD were screened for entries of identified variants. Quality filtering was done using built-in GSvar filters that could exclude variants with a depth under 140. In passing variants with low coverage or quality “Integrative Genomics Viewer” (IGV) was used for manual review of variants. Sequencing errors can be indicated by cluster of bases with low quality or read coverage and high rates of mismatches [1, 2].

Data analysis

*Variant classification*

The American College of Medical Genetics and Genomics (ACMG) standards for the interpretation of sequence variants were used to classify all reported single nucleotide variants as follows: class 5 (pathogenic, PV), class 4 (likely pathogenic, LPV), class 3 (variants of unknown significance, VUS), class 2 (likely benign) and class 1 (benign) [3, 4]. Variants that were classified as benign or likely benign were not reported in this study. The nomenclature guidelines of the Human Genome Variation Society (HGVS) were used to annotate DNA sequence variants [5]. By analyzing trio WES, data was filtered for *de novo* variants as well as homozygous, compound heterozygous, and hemizygous variants in affected patients but not in unaffected family members.

*Copy number analysis*

Copy number variant (CNV) analysis to detect disease-causing losses and gains was performed for all patients with non-confirmatory genetic diagnosis through targeted panel diagnostics (n=99). To interpret copy number variants (CNV), we followed the recommendations of Riggs *et al* [6]**.** CNV calling was performed using ClinCNV [7] based on the depth of coverage, i.e. the number of reads in an adjacent region as described elsewhere [8]. Rare copy number variants affecting OMIM genes were followed up in a similar fashion as the aforementioned single nucleotide variants (SNVs), small insertions, or deletions (indels).

Sanger sequencing

Segregation analysis for potential PVs was performed by Sanger sequencing. The according variants were amplified by PCR from the genomic DNA using primers (Supplemental Table S6) and conditions, which can be provided upon request. Amplicons were purified (MinElute 96 UF PCR Purification Kit, QIAGEN, Hilden, Germany) and cycle-sequenced using fluorescent dye-termination (BigDye Terminator v1.1 Cycle Sequencing Kit, Applied Biosystems, Darmstadt, Germany) and an ABI 3100 or ABI 310 automatic capillary genetic analyzer. Sequencing results were compared with the sequence obtained from WES.

WES analysis

Analysis of WES data was performed on patients with non-confirmatory genetic diagnosis through virtual panel diagnostics (vPANEL_1 and vPANEL_2). For additional evaluation of WES, the human phenotype ontology (HPO) annotation was used for deep phenotyping. The HPO terms used for variant filtering were “Autoimmunity” (HP:0002960, associated with 155 genes) and HPO terms according to the phenotype of every individual (Supplemental Table S7 and S8). In addition, genetic variants detected in the patients were filtered for the most likely deleterious variants, for example, nonsynonymous point mutations, losses/gains of one or several base pairs, splice variants, or microdeletions/microduplications (copy number variations [CNVs]).

Below, we describe the six patients with positive genetic results after vPANEL_2 screening.

Patient ID 23 suffered from severe pulmonary arterial hypertension with right heart failure due to diaphragmatic herniation resulting in lung hypoplasia. He underwent sequential double lung transplantation at age 5 years followed by a complicated course with multiple suspected acute rejections and chronic allograft dysfunction. As the patient showed signs of autoinflammatory symptoms whenever steroids were reduced, genetic testing was initiated. This identified the previously unreported hemizygous LPV c.1511G>C p.(Gly504Ala) in the *CYBB* gene. PVs of *CYBB* are responsible for the most common form of chronic granulomatous disease [9-11] (CGD, MIM306400), a primary immunodeficiency in which phagocytic cells display little or no NADPH oxidase activity [12, 13]. Flow cytometry assessing respiratory burst activity in phagocytes confirmed the CDG diagnosis. Based on the IUIS classification of immunological diseases, this disease belongs to the “Congenital Defects of Phagocyte Number or Function” (Table 5) or “Defects in Intrinsic and Innate Immunity” [14] (Table 6). In addition, using the WES_3 data analysis, we also identified a second LPV c.1276delG p.(Ala426Profs*45) in the *ZFPM2* gene. This variant has not been previously reported. Heterozygous PVs in *ZFPM2* cause congenital diaphragmatic hernias and structural anomalies of the diaphragm [15]. A reduced penetrance is recorded [16]. He finally passed away at age of 8 years due to terminal allograft dysfunction.

In patient ID26, two compound heterozygous LPVs were detected in the *TTC37* gene (c.2453_2454delTG p.(Val818Glufs*5) and c.(-228+1216_-228+1222)_(326+92_326+98)del p.?), leading to a diagnosis of tricho-hepato-enteric syndrome 1 [17]. The patient experienced the first symptoms at the age of 6 months; these included severe pneumonia followed by recurrent infection and persistent diarrhea in the following years. This patient nevertheless showed a mild phenotype with spontaneous remission of diarrhea, no hypogammaglobulinemia, and low antibody production after vaccination against pneumococcus. The IUIS classifies the underlying disease as “Combined Immunodeficiency with Associated or Syndromic Features” [14] (Table 2b).

In patient ID18, the identification of the homozygous PV c.477G>A p.(Trp159*) in the *IL10RB* gene confirmed the diagnosis of an IL-10-receptor defect [18]. The variant has been previously described in 6 other patients [18]. The parents of this patient are first cousins. The patient showed the first symptoms of autoinflammatory disease during the neonatal period. Symptoms included failure to thrive, recurrent episodes of fever, maculopapular skin lesions, intermittent bloody diarrhea, and hearing impairment. Allogeneic bone marrow transplantation led to remission. Based on the IUIS classification of immunological diseases, this disease belongs to “Diseases of Immune Dysregulation” [14] (Table 4).

In patient ID55, a heterozygous LPV was detected in the gene *MS4A1* (c.203dup p.(Lys9Valfs*14)). Biallelic PVs in *MS4A1* are known to cause CVID with hypogammaglobulinemia (MIM613495). Heterozygous carriers have been described to have impaired expression of CD20 on IgG4-expressing plasma cells [19]. Segregation analysis revealed that the healthy father as well as the deceased grandmother, who suffered from M. Ormond, were carriers as well. A variable penetrance can thus be assumed. Based on the IUIS classification of immunological diseases, this disease belongs to “Predominantly Antibody Deficiencies” [14] (Table 3).

Patient ID19 presented with classical symptoms of a periodic fever syndrome like limb pain, splenomegaly, and swollen joints with additional effusion later during the course of illness. Genetic testing identified a homozygous PV in the gene *ADA2* (c.139G>A p.(Gly47Arg)). Biallelic PVs in *ADA2* led to vasculitis with autoinflammation (MIM61588), explaining the phenotype observed in our patient [20, 21].

In patient ID25, WES identified a heterozygous PV in the gene *RIPK1* (c.970G>C p.(Asp324His)) as *de novo*. This variant was already described to cause cleavage-resistant RIPK1-induced autoinflammatory syndrome (CRIA-syndrome) [22, 23]. Our patient showed recurrent fever without a certain periodicity, hepatosplenomegaly, lymphadenitis, and microcytic anemia. Treatment with IL-6-inhibitors improved the symptoms.

**References**

(1) Robinson JT, Thorvaldsdóttir H, Winckler W, Guttman M, Lander ES, Getz G, et al. Integrative genomics viewer. Nat Biotechnol 2011 Jan;29(1):24-26.

(2) Robinson JT, Thorvaldsdóttir H, Wenger AM, Zehir A, Mesirov JP. Variant Review with the Integrative Genomics Viewer. Cancer Res 2017 Nov 1;77(21):e31-e34.

(3) Richards S, Aziz N, Bale S, Bick D, Das S, Gastier-Foster J, et al. Standards and guidelines for the interpretation of sequence variants: a joint consensus recommendation of the American College of Medical Genetics and Genomics and the Association for Molecular Pathology. Genet Med 2015 May;17(5):405-424.

(4) Abou Tayoun AN, Pesaran T, DiStefano MT, Oza A, Rehm HL, Biesecker LG, et al. Recommendations for interpreting the loss of function PVS1 ACMG/AMP variant criterion. Hum Mutat 2018 Nov;39(11):1517-1524.

(5) den Dunnen JT, Dalgleish R, Maglott DR, Hart RK, Greenblatt MS, McGowan-Jordan J, et al. HGVS Recommendations for the Description of Sequence Variants: 2016 Update. Hum Mutat 2016 Jun;37(6):564-569.

(6) Riggs ER, Andersen EF, Cherry AM, Kantarci S, Kearney H, Patel A, et al. Technical standards for the interpretation and reporting of constitutional copy-number variants: a joint consensus recommendation of the American College of Medical Genetics and Genomics (ACMG) and the Clinical Genome Resource (ClinGen). Genet Med 2020 Feb;22(2):245-257.

(7) Tangye SG, Al-Herz W, Bousfiha A, Chatila T, Cunningham-Rundles C, Etzioni A, et al. Human Inborn Errors of Immunity: 2019 Update on the Classification from the International Union of Immunological Societies Expert Committee. J Clin Immunol 2020 Jan;40(1):24-64.

(8) Demidov G, Ossowski S. ClinCNV: novel method for allele-specific somatic copy-number alterations detection. bioRxiv 2019:837971.

(9) Roos D. Chronic granulomatous disease. Br Med Bull 2016 Jun;118(1):50-63.

(10) Bustamante J, Arias AA, Vogt G, Picard C, Galicia LB, Prando C, et al. Germline CYBB mutations that selectively affect macrophages in kindreds with X-linked predisposition to tuberculous mycobacterial disease. Nat Immunol 2011 Mar;12(3):213-221.

(11) Bustamante J, Picard C, Fieschi C, Filipe-Santos O, Feinberg J, Perronne C, et al. A novel X-linked recessive form of Mendelian susceptibility to mycobaterial disease. J Med Genet 2007 Feb;44(2):e65.

(12) Roos D, Kuhns DB, Maddalena A, Roesler J, Lopez JA, Ariga T, et al. Hematologically important mutations: X-linked chronic granulomatous disease (third update). Blood Cells Mol Dis 2010 Oct 15;45(3):246-265.

(13) Winkelstein JA, Marino MC, Johnston RB,Jr, Boyle J, Curnutte J, Gallin JI, et al. Chronic granulomatous disease. Report on a national registry of 368 patients. Medicine (Baltimore) 2000 May;79(3):155-169.

(14) Bousfiha A, Jeddane L, Picard C, Al-Herz W, Ailal F, Chatila T, et al. Human Inborn Errors of Immunity: 2019 Update of the IUIS Phenotypical Classification. J Clin Immunol 2020 Jan;40(1):66-81.

(15) Brady PD, Van Houdt J, Callewaert B, Deprest J, Devriendt K, Vermeesch JR. Exome sequencing identifies ZFPM2 as a cause of familial isolated congenital diaphragmatic hernia and possibly cardiovascular malformations. Eur J Med Genet 2014;57(6):247-252.

(16) Longoni M, Russell MK, High FA, Darvishi K, Maalouf FI, Kashani A, et al. Prevalence and penetrance of ZFPM2 mutations and deletions causing congenital diaphragmatic hernia. Clin Genet 2015 Apr;87(4):362-367.

(17) Fabre A, Bourgeois P, Chaix C, Bertaux K, Goulet O, Badens C. Trichohepatoenteric Syndrome. 2018 Jan 11. In: Adam MP, Ardinger HH, Pagon RA, Wallace SE, Bean LJH, Gripp KW, Mirzaa GM, Amemiya A, editors. GeneReviews^®^ [Internet]. Seattle (WA): University of Washington, Seattle; 1993–2022.

(18) Glocker EO, Kotlarz D, Boztug K, Gertz EM, Schäffer AA, Noyan F, et al. Inflammatory bowel disease and mutations affecting the interleukin-10 receptor. N Engl J Med 2009 Nov 19;361(21):2033-2045.

(19) Grimm KE, Bakke A, O'Malley DP. Abnormal expression of CD20 on IgG4 plasma cells associated with IgG4-related lymphadenopathy. Arch Pathol Lab Med 2013 Sep;137(9):1282-1285.

(20) Lee PY. Vasculopathy, Immunodeficiency, and Bone Marrow Failure: The Intriguing Syndrome Caused by Deficiency of Adenosine Deaminase 2. Front Pediatr 2018 Oct 18;6:282.

(21) Zhou Q, Yang D, Ombrello AK, Zavialov AV, Toro C, Zavialov AV, et al. Early-onset stroke and vasculopathy associated with mutations in ADA2. N Engl J Med 2014 Mar 6;370(10):911-920.

(22) Tao P, Sun J, Wu Z, Wang S, Wang J, Li W, et al. A dominant autoinflammatory disease caused by non-cleavable variants of RIPK1. Nature 2020 Jan;577(7788):109-114.

(23) Lalaoui N, Boyden SE, Oda H, Wood GM, Stone DL, Chau D, et al. Mutations that prevent caspase cleavage of RIPK1 cause autoinflammatory disease. Nature 2020 Jan;577(7788):103-108.
